# Omega-3 fatty acids accelerate fledging in an avian marine predator: a potential role of cognition

**DOI:** 10.1242/jeb.235929

**Published:** 2021-02-24

**Authors:** Jessika Lamarre, Sukhinder Kaur Cheema, Gregory J. Robertson, David R. Wilson

**Affiliations:** 1 Cognitive and Behavioural Ecology Program, Memorial University of Newfoundland, St John's, NL, Canada, A1B 3X9; 2 Department of Biochemistry, Memorial University of Newfoundland, St John's, NL, Canada, A1B 3X9; 3 Wildlife Research Division, Environment and Climate Change Canada, Mount Pearl, NL, Canada, A1N 4T3; 4 Department of Psychology, Memorial University of Newfoundland, St John's, NL, Canada, A1B 3X9

**Keywords:** Bird, Brain development, Essential fatty acid, Docosahexaenoic acid, Eicosapentaenoic acid, Aquatic ecosystem

## Abstract

Consuming omega-3 fatty acids (n-3 LCPUFAs) during development improves cognition in mammals, but the effect remains untested in other taxa. In aquatic ecosystems, n-3 LCPUFAs are produced by phytoplankton and bioaccumulate in the food web. Alarmingly, the warming and acidification of aquatic systems caused by climate change impair n-3 LCPUFA production, with an anticipated decrease of 80% by the year 2100. We tested whether n-3 LCPUFA consumption affects the physiology, morphology, behaviour and cognition of the chicks of a top marine predator, the ring-billed gull. Using a colony with little access to n-3 LCPUFAs, we supplemented siblings from 22 fenced nests with contrasting treatments from hatching until fledging; one sibling received n-3 LCPUFA-rich fish oil and the other, a control sucrose solution without n-3 LCPUFAs. Halfway through the nestling period, half the chicks receiving fish oil were switched to the sucrose solution to test whether n-3 LCPUFA intake remains crucial past the main growth phase (chronic versus transient treatments). Upon fledging, n-3 LCPUFAs were elevated in the blood and brains of chicks receiving the chronic treatment, but were comparable to control levels among those receiving the transient treatment. Across the entire sample, chicks with elevated n-3 LCPUFAs in their tissues fledged earlier despite their morphology and activity levels being unrelated to fledging age. Fledging required chicks to escape fences encircling their nest. We therefore interpret fledging age as a possible indicator of cognition, with chicks with improved cognition fledging earlier. These results provide insight into whether declining dietary n-3 LCPUFAs will compromise top predators' problem-solving skills, and thus their ability to survive in a rapidly changing world.

## Abstract (precis)

**Summary:** Supplementing gull chicks with omega-3 fatty acids in the context of natural deficiency improves their cognitive development, as shown by their ability to fledge earlier by escaping a fence encircling their nest.

## INTRODUCTION

Obtaining omega-3 long-chain polyunsaturated fatty acids (n-3 LCPUFAs) during development is critical for cognition in mammals (Lozada et al., 2017; Mulder et al., 2014), and for proper physical growth and immune system function in vertebrates generally (Bauer et al., 2014; Calder, 2015; Castro et al., 2016; Dyall, 2015; Pappas et al., 2007; Speake and Wood, 2005). The main n-3 LCPUFAs providing these benefits are eicosapentaenoic acid (EPA) and docosahexaenoic acid (DHA; Hixson et al., 2015), which are concentrated in neuronal and retinal tissues (Janssen et al., 2015; Saini and Keum, 2018; Weiser et al., 2016). Although DHA has a structural and signalling role in neuronal membranes, whereas EPA is converted rapidly into other metabolites (primarily eicosanoids and DHA; Anderson et al., 1989; Chen et al., 2013; Kaur et al., 2010; McNamara and Carlson, 2006), both n-3 LCPUFAs are thought to benefit cognition through their neurogenesis and anti-inflammatory properties (Bazinet and Layé, 2014; Calder, 2015; Hoffman et al., 2009). During early development, n-3 LCPUFAs provide precursors for myelin sheath production (Feltham et al., 2019), thus ensuring the synaptic health and plasticity of the brain (review by Yang et al., 2018). Through their anti-inflammatory actions, they also maintain high blood flow to the brain (Bazinet and Layé, 2014; Calder, 2015). In humans and rodents, a deficiency of n-3 LCPUFAs during pregnancy and early development impairs the development of synaptic plasticity (Dyall, 2015; Wu et al., 2008), spatial memory (Delpech et al., 2015; Gamoh et al., 2011), early information processing and comprehension (Hoffman et al., 2009; Mulder et al., 2014), reaction time and functional activation of the cortex (Bauer et al., 2014; Li et al., 2018), and overall cognitive abilities (Bazinet and Layé, 2014; Chaung et al., 2013; Joffre et al., 2014). Although meta-analyses have clearly established the cognitive benefits of n-3 LCPUFAs in humans and non-human mammals (Mazereeuw et al., 2012; Jiao et al., 2014; Emery et al., 2020; Hooijmans et al., 2012), such benefits remain untested in other taxonomic groups. In birds, for example, n-3 LCPUFAs accumulate rapidly in the brains of nestlings (Price et al., 2018; Speake and Wood, 2005; Twining et al., 2018a), yet it remains unknown whether dietary restriction of n-3 LCPUFAs impairs avian cognition.

The availability of n-3 LCPUFAs differs markedly between terrestrial and aquatic systems as a result of differences in their resident primary producers (Colombo et al., 2016; Gladyshev et al., 2016; Hixson et al., 2015; Parrish, 2013). Terrestrial plants produce α-linolenic acid (ALA), an essential fatty acid and n-3 LCPUFA precursor, but do not synthesize EPA and DHA (Calder, 2015; Colombo et al., 2016; Heinze et al., 2012). Strict terrestrial herbivores convert ALA from plants into EPA and DHA (Hixson et al., 2015; Parrish, 2013; Twining et al., 2019), and terrestrial predators obtain n-3 LCPUFAs by consuming herbivores. The availability of n-3 LCPUFAs on land is thus restricted by limited accumulation in primary consumers (Colombo et al., 2016). In contrast, EPA and DHA are abundant in aquatic systems because they are produced by phytoplankton and bioaccumulate widely in zooplankton and small fish (Barrett et al., 2007; Colombo et al., 2016; Kainz et al., 2004). High-order predators in both ecosystems may be unable to efficiently synthesize n-3 LCPUFAs and thus depend on dietary consumption, especially during the critical period of early brain development (Gladyshev et al., 2016; Speake and Wood, 2005).

Alarmingly, the warming and acidification of aquatic ecosystems caused by pollution and the rising concentration of greenhouse gas emissions (Arts, 2002; Bækken et al., 2006; Doney, 2010; Pachauri et al., 2014) is impairing phytoplankton production of n-3 LCPUFAs (Colombo et al., 2016; Hixson and Arts, 2016; Meyers et al., 2019; Rossoll et al., 2012). Phytoplankton use n-3 LCPUFAs to control the fluidity of their membranes, but rising water temperatures cause them to replace their relatively fluid n-3 LCPUFAs with other, more rigid, fatty acids that have shorter and more-saturated carbon chains (Colombo et al., 2016, 2020; Hixson and Arts, 2016; Kang, 2011). In addition, water acidification is impeding nitrification necessary for rendering nitrogen biologically available to aquatic primary producers (Beman et al., 2011; Li et al., 2012; Rossoll et al., 2012). Nitrogen is the main limiting nutrient of phytoplankton and is required to synthesize the enzymes that facilitate n-3 LCPUFA production (Minhas et al., 2016; Schüler et al., 2017). Recent models predict that production of n-3 LCPUFAs in aquatic ecosystems will decline by more than 80% by the year 2100, relative to current levels (Bermudez et al., 2016; Colombo et al., 2020). This decline would nearly eliminate dietary access to n-3 LCPUFAs, particularly at higher trophic levels where bioaccumulation normally concentrates them in the tissues of fatty fishes, such as smelt (family Osmeridae), sandlance (family Ammodytidae), mackerel (family Scombridae), salmon (family Salmonidae), herring, sardines and sprat (family Clupeidae; Dave and Routray, 2018; Newton and Snyder, 1997). This bottom-up trophic cascade could compromise the health and cognition of top aquatic predators that depend on fatty fishes for n-3 LCPUFAs (Colombo et al., 2020; Gladyshev et al., 2016; Montevecchi, 2002; Speake and Wood, 2005).

Seabirds are top marine predators that may be especially vulnerable to the impacts of ocean warming and acidification. They forage primarily on zooplankton and fatty fishes, and, based on research on other top predators (Calder, 2015; Castro et al., 2012; Twining et al., 2018a), are unlikely to efficiently synthesize n-3 LCPUFAs *de novo*. Studies of opportunistic seabirds that have shifted their diets towards terrestrial foods containing little to no n-3 LCPUFAs (Caron-Beaudoin et al., 2013; Marteinson et al., 2015; Seif et al., 2018) provide some insight into the possible impacts of future declines in marine sources of dietary n-3 LCPUFAs. Populations that shifted their traditional aquatic diets to include both aquatic and terrestrial foods experienced increased breeding success (Auman et al., 2008; Lenzi et al., 2019; Weiser and Powell, 2010), but those that completely replaced their aquatic diets with terrestrial foods experienced decreased brood size, increased nestling mortality and slower chick growth (O'Hanlon et al., 2017; Pierotti and Annett, 2001; Sotillo et al., 2019). These studies compared the effects of terrestrial versus aquatic diets on avian reproduction, but did not specifically consider the potential cognitive consequences of decreased dietary n-3 LCPUFAs that likely accompanied the shift to a terrestrial diet.

In the current study, we tested two separate hypotheses. First, we tested whether increasing the consumption of n-3 LCPUFAs elevates the concentration of n-3 LCPUFAs in the tissues of developing chicks in a top marine predator, the ring-billed gull (*Larus delawarensis*). We used a population of ring-billed gulls that has been reported as foraging only in terrestrial habitats (city, landfill, fields) and not consuming fish or other foods that contain n-3 LCPUFAs (Aponte et al., 2014; Brousseau et al., 1996; Caron-Beaudoin et al., 2013; Marteinson et al., 2015). Although a positive relationship between n-3 LCPUFAs in the diet and n-3 LCPUFAs in the tissues is expected (e.g. Heinze et al., 2012; Price et al., 2018; Twining et al., 2016), it is also possible that this relationship is disrupted by the transfer of n-3 LCPUFAs from the yolk to the chick (Pappas et al., 2007; Speake and Wood, 2005; Surai et al., 2001), or by chicks synthesizing n-3 LCPUFAs from the n-3 LCPUFA precursor. Thus, for our second hypothesis, we tested whether a greater concentration of n-3 LCPUFAs in the chicks' tissues would improve their cognitive abilities, regardless of the source of this increase.

During late-stage incubation, we encircled 30 nests with individual fences designed to contain chicks after hatching. Following hatching, we supplemented the chicks throughout the nestling period with fish oil rich in n-3 LCPUFAs or with a control sucrose solution as a caloric equivalent devoid of n-3 LCPUFAs, and then tested whether these supplements had effectively manipulated n-3 LCPUFAs in blood and brain tissue. Then, near the end of the nestling period, we attempted to test the chicks' cognition using standard string pull tests (Danel et al., 2019; Heinrich and Bugnyar, 2005; Jacobs and Osvath, 2015), but, unfortunately, the chicks were not motivated to solve them. We realized, however, that chicks could only fledge by flying over the fence encircling their nest, and that the age of fledging might therefore reflect their ability to solve problems. Our logic derives from earlier research on cognition, where escaping more quickly from a puzzle box was interpreted as evidence of enhanced cognition (Cauchard et al., 2013; Galsworthy et al., 2005; Grundy et al., 2014). Of course, differences in physical growth rates and overall activity levels could also influence fledging age. We therefore quantified growth rate and activity levels to determine whether these were related to fledging age. If they were not, then we would interpret earlier fledging by chicks with more n-3 LCPUFAs in their tissues as evidence that n-3 LCPUFAs improve cognition.

## MATERIALS AND METHODS

### Experimental model and subject details

**Fig. 1.**
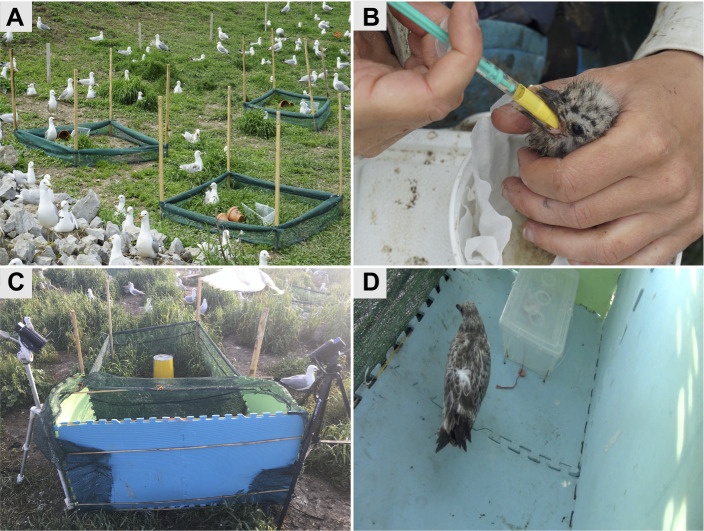
**Photographs of the fenced nests, gavage procedure and string-pull test apparatus.** (A) Fences deployed around nests were initially at a height of 15 cm above the ground and were raised gradually to 90 cm thereafter. An open string-pull task box allowed chicks to habituate to the test apparatus, and two clay planter pots provided hiding places. (B) Gavage of chicks using a syringe. The same technique was used for fish oil and sucrose solution supplementations. (C) The enclosure used for the string-pull test in relation to the nest of the chick being tested. The front of the enclosure was semi-transparent to permit interaction with the parents at the nest. While one sibling was undergoing a trial, other siblings were kept under a meshed container (yellow) that allowed communication with the parents but prevented the siblings from viewing the trial. Two cameras recorded each trial and foam mats (not pictured) were laid on top of the cameras during a trial to prevent gulls from flying into the enclosure. (D) Chick in front of the string-pull box while undergoing the string-pull test. Two sausage pieces were laid on either side of the string and a third piece was left in the box, accessible only by pulling on the string.

From 15 to 21 May 2019, at the end of the incubation period, 30 ring-billed gull (*Larus delawarensis* Ord 1815) nests were selected within 1–20 m of the periphery of the Beauharnois breeding colony near Montreal, Canada (45°18′58.6″N 73°54′22.6″W). Nests were selected haphazardly, but with the constraints that they contained three unhatched eggs and were not within 1.5 m of another nest. We contained chicks that eventually hatched by constructing a fence around each selected nest; four wooden posts (2.5×5.1×122 cm) were inserted partially into the ground in a square arrangement (1.3×1.3 m) centred on the nest, and semi-transparent synthetic burlap (90 cm height) was then wrapped around the posts, stapled to them, and fastened to the ground with tent pegs (Fig. 1A). At construction, the burlap was rolled onto itself from the top to stand at a height of only 15 cm from the ground to reduce visual disturbance at the site and encourage parents to resume incubation quickly. All nests were visually monitored for parental abandonment, but all parents resumed incubation within seconds of the researchers departing their immediate nest area. The height of the burlap fence was increased gradually after hatching to prevent the growing chicks from jumping over. Once chicks reached 23 days old, the burlap stood at its full height of 90 cm and chicks could only escape by flying over it.

We attempted to maintain our sample size at 30 nests. If a clutch failed to hatch or all chicks died before fledging, it was removed from the experiment and another nest containing three unhatched eggs was added to the experiment until no such nests could be found at the colony. Early during our study, 28 chicks (15 in the control treatment and 13 in the fish oil treatment) died before reaching 10 days of age, mainly as a result of inclement weather. Thus, we attempted to replace them by targeting new, unhatched clutches. Our final sample size included 36 chicks from 22 nests. However, two chicks from the sucrose control group were attacked and found dead in their nest at 38 days post-hatching (dph), before they had fledged (see Fig. S1 for sample size and hatching date details). Chicks were monitored daily from hatching until fledging (maximum 42 dph). We considered 42 days old as the maximum fledging age of this population as previous studies indicated that ring-billed gulls nesting in an urban environment would fledge between 30 and 42 days old (Chardine, 1978; Mousseau, 1984; Pollet et al., 2012). All methods were performed under appropriate permits (Canadian Wildlife Service Scientific Permit, number SC-80; Environment and Climate Change Canada Scientific Permit to Capture and Band Migratory Birds, numbers 10890 and 10890B; Letter of Authorization from the Ministère de l'Environnement et de la Lutte contre les changements climatiques to conduct research on the Beauharnois lot #4716939) and were approved by the Memorial University Animal Care Committee (number 19-03-DW). We minimized disturbance to the colony by using nests near the periphery, remaining inside a blind when not at a nest, and minimizing the time spent at the colony.

### Identification

Nests were monitored daily for evidence of hatching. Immediately after a chick had hatched and was dry, it was captured by hand and identified by colouring its right or left axilla green or black with a non-toxic marker (colour and side randomly selected by coin-toss, but constrained to have different identification between siblings). The marker was reapplied daily until 6–7 dph, when their right tarsometatarso-phalangial joint was thick enough to hold a temporary colour band (green, blue or mint, randomly selected) that expands with growth. At 15 days old, past the main mortality period of the first 2 weeks post-hatching (Chardine, 1978), the chicks were banded with a permanent and uniquely numbered Canadian Wildlife Service band on their left leg. Hatching date, hatching order and fledging date were recorded for all chicks (we define hatching day as 1 dph), though hatching order could not be determined for five nests because the first two eggs in each nest hatched during the same night.

### Supplementation

The first chick to hatch in a nest was assigned to either a control group receiving a sucrose solution (no fatty acids) or an experimental group receiving Menhaden fish oil (Table S1; Sigma-Aldrich), rich in n-3 LCPUFAs. Although fish oil contains other fatty acids besides EPA and DHA (Table S1), previous research has established that only its n-3 LCPUFA content affects cognition (e.g. Bauer et al., 2014; Bazinet and Layé, 2014; Mazereeuw et al., 2012; Vinot et al., 2011; Zhang et al., 2016). Nevertheless, we acknowledge that our dietary supplements differ in more ways than just the presence versus absence of n-3 LCPUFAs. Block randomization was used to assign chicks to treatments to ensure that hatching order was balanced across dietary groups. Thus, chicks that hatched second received the opposite treatment from their older sibling. We only had one instance of a third-hatched chick surviving beyond 10 days old. It was assigned to the fish oil treatment. In addition to the supplementation, chicks were fed naturally by their parents, though we could not quantify how much food the parents provided.

We chose to make the control solution with sucrose as opposed to a type of fat that does not contain n-3 LCPUFAs for two reasons. First, we required the control solution to remain liquid at room temperature, as is the case for fish oil, to facilitate gavage. This excluded all types of saturated fats, including shortening, coconut oil and beef tallow. Second, we required a supplementation that contained neither ALA nor linoleic acid, thus ruling out all plant-based oils (De Lorgeril et al., 2001; Dubois et al., 2007; Orsavova et al., 2015). ALA is a precursor for EPA and DHA; although we would not expect aquatic predators such as ring-billed gulls to efficiently convert ALA into n-3 LCPUFAs (Gladyshev et al., 2016; Speake and Wood, 2005; Twining et al., 2018a), we could not rule out this possibility. Moreover, if ring-billed gulls have indeed conserved this ability, the conversion of ALA into n-3 LCPUFAs would have been energetically costly (Brenna and Carlson, 2014), and would thus confound the effects of dietary n-3 LCPUFAs on development (Twining et al., 2019). Conversely, linoleic acid is the omega-6 fatty acid precursor of arachidonic acid (AA) and both compete metabolically with n-3 LCPUFAs (Saini and Keum, 2018). Using a control supplementation containing linoleic acid could have counteracted the natural accretion of n-3 LCPUFAs in tissues (Brenna et al., 2009; Hibbeln et al., 2006). We chose sucrose for our control because it is readily digested and absorbed by a wide range of avian omnivores (common grackle, *Quiscalus quiscula*: Martinez del Rio et al., 1988; red-winged blackbird, *Agelaius phoeniceus*: Martinez del Rio et al., 1988; azure-winged magpie, *Cyanopica cyanus*: Lane, 1997; cape white-eye: *Zosterops virens*: Wellmann and Downs, 2009; dark-capped bulbul, *Pycnonotus tricolor*: Brown et al., 2010; house sparrow, *Passer domesticus*: Rott et al., 2017; mallard, *Anas platyrhynchos*: Kohl et al., 2017; quail, *Coturnix coturnix*: Kohl et al., 2017), and because difficulties with sucrose digestion have only been documented in the families Sturnidae, Turdidae and Mimidae (Brown et al., 2012; Gatica et al., 2006). Nevertheless, we acknowledge that the macronutrient content of sucrose and fish oil do not overlap, which could influence our results beyond the simple addition of n-3 LCPUFAs to our subjects' diet.

The quantity of fish oil provided daily to experimental chicks was based on the maximum amount of n-3 LCPUFAs that ring-billed gull chicks in a marine population consume at a given age. Menhaden fish oil has a similar ratio of EPA:DHA to Alewife (*Pomolobus pseudoharengus*; USDA, 2019), a common prey of ring-billed gulls (Haymes and Blokpoel, 1978; Jarvis and Southern, 1976; Pollet et al., 2012). Although considerable variation exists among populations, the diet of ring-billed gulls comprises a maximum of 80% marine-derived food, including alewife (Brousseau et al., 1996; Kirkham and Morris, 1979; Washburn et al., 2013). We therefore supplemented experimental chicks with a quantity of fish oil equivalent to the amount of n-3 LCPUFAs that they would consume from a diet comprising 80% alewife (Table S2). Chicks in our population are unlikely to consume significant amounts of n-3 LCPUFAs in their diet (Brousseau et al., 1996; Caron-Beaudoin et al., 2013), so the total amount of n-3 LCPUFAs consumed by experimental chicks should remain within the natural range for this species. Age-specific masses were estimated in advance according to previous studies (Chardine, 1978; Dawson et al., 1976; Iacovides and Evans, 1998; Oswald et al., 2013) so that supplements could be prepared. Total energetic requirements at each age were calculated according to the age-specific and mass-specific formula for the closely related herring gull, *Larus argentatus* (mass: Drent et al., 1992; Dunn, 1976; Norstrom et al., 1986; phylogeny: Liebers-Helbig et al., 2010; Pons et al., 2005), as a comparable formula does not exist for ring-billed gulls. Herring gulls and ring-billed gulls have similar distributions, lay dates, incubation time, brood size, fledging age and growth patterns (Nisbet et al., 2017; Pollet et al., 2012). We therefore assumed that these two species required similar energy intake per unit of mass per age. The sucrose control comprised white sugar dissolved in a volume of water equal to the volume of oil provided to experimental chicks of the same age, and the two treatments had identical caloric content.

From hatching until fledging (maximum 42 dph), chicks were supplemented daily at the nest by gavage using a 1 or 3 ml syringe (Fig. 1B). Because n-3 LCPUFA accretion in the brain is thought to subside after the main linear growth period is achieved (Speake and Wood, 2005; Speake et al., 2003), the fish oil treatment was subdivided into two groups at day 22: a chronic group that remained supplemented with fish oil until fledging and a transient group that was switched to sucrose until fledging. This transient treatment tested whether there is a cumulative benefit of consuming n-3 LCPUFAs after the first 3 weeks post-hatching, once ring-billed gulls have completed their main growth phase (Drent et al., 1992; Iacovides and Evans, 1998; Pollet et al., 2012). The only third-hatched chick in our sample was assigned to the chronic group, opposite to that of its older sibling that had been randomly assigned to the transient group. The other sibling in the nest remained on sucrose.

### Morphology and tissue samples

Body measurements were taken daily from hatching until 22 dph, and weekly thereafter until fledging. Chicks were weighed on a digital scale (smart weigh TOP500; lot no. A14-336; accuracy: ±0.01 g), and culmen and tarsus length were measured with a calliper (accuracy: ±0.05 mm). At 15 and 36 dph (representing 2 weeks after the supplementation was initiated and 2 weeks after the transient group's supplementation was switched from fish oil to the control solution, respectively), blood samples were drawn from the brachial vein using a hypodermic syringe to ensure accretion of n-3 LCPUFA into the birds' tissue in accordance with their treatment group. One of the two chronic oil group chicks that fledged at 37 dph could not be recaptured for a blood sample at 36 dph because heavy rain restricted access to the colony on that day. The blood was collected in 600 μl lithium–heparin-coated BD Microtainers with a plasma separator (BD; cat. no. B365985) to prevent blood coagulation prior to plasma separation. Blood samples were kept on ice in the field for less than 12 h before being centrifuged at 2000 ***g*** for 4 min to separate the plasma from the cell fraction. After centrifugation, the plasma phase was transferred into a new tube and both plasma and cell fraction phases were stored at −20°C until further analysis.

Twelve birds (four per treatment) were randomly selected and killed by cervical dislocation at 42 days old to analyse the fatty acid profiles of their cerebral hemispheres and determine whether the levels of n-3 LCPUFAs in their blood reflected an accretion in their brain, the latter being a predictor of enhanced cognition (Bazinet and Layé, 2014; Luchtman and Song, 2013). The carcasses were immediately placed on ice in the field and were stored at −80°C within 4 h of death. Carcasses and blood samples were flown on ice in a refrigerated (4°C) cargo hold on a plane to Memorial University of Newfoundland and stored at −20°C thereafter.

### Behaviours during the string-pull test

A modified version of the horizontal string-pull task (Danel et al., 2019; Jacobs and Osvath, 2015) was designed to test the chicks' problem-solving skills through their ability to access a treat inside a clear plastic box by pulling repeatedly on a string (Fig. 1D). Although chicks ultimately showed little interest in the string-pull task, our video recordings of the trials allowed us to quantify their behaviour and overall activity levels. From 23 days old until fledging (maximum 42 dph), each chick was tested individually every other day. Testing order changed each day and was selected to minimize disturbance to the colony and to parental provisioning. Prior to each test, an enclosure (1.2×0.6×0.6 m L×W×H; Fig. 1C) with opaque walls on three sides and a semi-transparent wall on the fourth side was placed adjacent to the fence surrounding the nest, such that the semi-transparent side of the enclosure pressed against the semi-transparent fencing. We provided visual contact between the enclosure and the fenced area around the nest because preliminary trials on chicks not involved in our study showed that parents produced alarm calls that elicited hiding responses in their chicks when visual contact between the two was lost. The string-pull task box was placed inside the enclosure and contained a piece of sausage (5 g) that could be accessed by pulling horizontally on a red string extending out of the box (Fig. 1D). To encourage the chicks to investigate the string, two more pieces of sausage were left outside the box on either side of it.

A trial began by placing the subject inside the enclosure and ended 15 min later. Researchers remained outside the colony during the trial, but the enclosure was video-recorded throughout with two cameras (Canon VIXIA HF R800; 1920×1080 resolution, 35mbps using MP4 compression, 60 frames s^−1^) covering all angles (Fig. 1C). Trials were conducted prior to daily supplementation and any morphological measures or blood samples. To prevent social learning, the siblings of the chick being tested were kept beneath a meshed receptacle that blocked their view of the enclosure during their sibling's trial (Fig. 1C). The parents could still see and communicate with all of their chicks during all trials to reduce the risk of nest abandonment. Once the trial was over, the chick was returned to its nest, its siblings were released from the mesh receptacle, the enclosure was removed from the nesting area, and supplementation was resumed.

### Video analysis

Using the software BORIS (version 7.9 RC1; Friard and Gamba, 2016), we analysed chick behaviours from the video recordings of the string-pull task. For each trial, we analysed the period between 3 and 13 min of the 15 min trial to avoid potential disturbances caused by the researchers departing and approaching the test apparatus at the start and end of the trial. Any time spent outside of the recording frame was removed from the total recording time (600 s) to account only for the actual recording time where behaviours could be measured. We counted the number of steps (each time the right foot lifted from the ground), divided by the actual recording time, and measured the percentage of actual recording time spent in contact with the fence separating the chick from its nest. We also noted any attempts to jump out of the enclosure, but these were too rare to be analysed statistically. Although our hypothesis is that n-3 LCPUFAs improve cognition, which, in turn, allows chicks to fledge earlier, it is also possible that the fish oil supplementation accelerated growth or increased activity levels through effects on muscle aerobic performance (Guglielmo, 2010; Maillet and Weber, 2007; Nagahuedi et al., 2009), which led to chicks fledging earlier. Our regular measurement of morphology and activity levels therefore allowed us to distinguish among these three hypotheses.

### Fatty acid analysis

We chose to analyse the fatty acid composition of the cerebral hemispheres because they are responsible for higher cognitive functions (Güntürkün, 2005; Nieder, 2017). We also analysed the fatty acid composition of red blood cells (RBCs), which have a 2 week turnover rate (Bearhop et al., 2002) and which should therefore reflect fatty acid consumption over a substantial portion of the 42 day nestling period. In contrast, blood plasma only reflects the nutrients absorbed in the previous 24–48 h (Hobson and Clark, 1993) and can be affected unpredictably by endogenous processes (Stark et al., 2016), making it less reliable than RBCs as a dietary marker.

The cerebral hemispheres were dissected out of the frozen skulls and flash-frozen with liquid nitrogen before being pulverized and homogenized with a mortar and pestle (Balogun et al., 2013). Total lipids were extracted from 300 μl of the RBC fraction and from a 35 mg subsample of the cerebral hemisphere according to the method of Folch et al. (1957). Transmethylation and organic extraction were performed according to the method of Chechi et al. (2010). The organic layer was then dried under nitrogen, dissolved in 50 ml of carbon disulfide, and run in the gas chromatograph for 45 min on an Omegawax×320 (30 m×0.32 mm) column from Supelco (Sigma-Aldrich) using a flame ionization detector (Chechi et al., 2010). Fatty acid standards (PUFA-2, PUFA-3 and Supelco 37 component FAME mix; Sigma-Aldrich) were used for identification of fatty acids by retention time. A non-naturally occurring internal standard (nonadecanoic acid C19:0, Sigma-Aldrich) of known concentration was added to each sample prior to transmethylation and used to derive the concentration of each fatty acid. The same fatty acid profile was quantified for both tissues to facilitate the comparison between the fatty acid levels of RBCs and brain tissue. The results are expressed as relative concentration (percentage of total identified fatty acids).

### Statistical analysis

All statistical analyses were performed in R, version 3.6.1 (http://www.R-project.org/). All models were validated using diagnostic plots of residuals versus fitted values and qqplots to ensure that there were no patterns observed in the residuals and that they were normally distributed. When mixed models were used, the distribution of the random effect was plotted to ensure it met the assumption of normality. We also simulated the responses of all models and plotted the simulations against the raw data to ensure an appropriate overlap between the two. Only statistically significant interactions were kept, otherwise they were dropped and the model refitted. Significance thresholds were set at *P*<0.05.

Nest identity and hatching order were not included as random or fixed effects in the final statistical models. Because of natural mortality, half of the nests in our final sample fledged only a single chick, and the lack of siblings at fledging caused convergence problems in preliminary models containing nest identity as a random effect. Furthermore, among the 13 nests where siblings fledged, none of the measured variables (fledging age, EPA, DHA, ALA, AA, mass gain, number of steps taken or time spent near the fence) were correlated between the first-hatched and second-hatched siblings (Pearson correlation: all *n*=13, all *r*<0.3, all *P*>0.05). Hatching order was excluded because, in five nests, the first two chicks hatched overnight and could not be assigned to a hatching order. Furthermore, many studies show that first-hatched and second-hatched gulls do not differ in terms of hatching success, morphology and fledging success (Bolton, 1991; Bosman, 2014; Brown, 1998; Chardine, 1978; Nisbet and Drury, 1972; Royle and Hamer, 1998).

#### Incorporation of n-3 LCPUFAs into blood and brain tissue

To test the effect of dietary treatment (sucrose solution control, transient oil and chronic oil) and chick age (15 and 36 dph) on the concentration of fatty acids in RBCs, we conducted a linear mixed-effect model for each fatty acid response (EPA, DHA). Chick identification was included as a random effect to account for the fact that the same chicks were measured at 15 and 36 dph. Tukey *post hoc* tests were performed to investigate where the differences occurred among the three treatment levels.

A linear model was used to test the relationship between DHA in RBCs at 36 dph (continuous predictor variable) and DHA in the cerebral hemispheres at 42 dph (continuous response variable). The same analysis was conducted for EPA.

#### n-3 LCPUFAs and early fledging

Fledging was recorded as the day when a chick was first found outside of the fencing surrounding its nest. Based on previous research and our own observations, the maximum fledging age of ring-billed gulls nesting in the greater Montreal area is 42 dph (Mousseau, 1984). We transformed fledging age by calculating the number of days before reaching 42 dph when the chick was found outside of its fenced nest for the first time. This resulted in an integer response variable (number of whole days) with a range of 0 to 8 days early, where 0 indicates that the chick had not left the nest before reaching the fledging age of 42 dph. We transformed fledging age in this fashion to create a positively skewed response variable that was amenable to statistical modelling. We tested the effect of DHA and EPA concentrations in RBCs at 36 dph (continuous predictors) on fledging age using a generalized linear model with a negative binomial distribution to account for overdispersion. A second generalized linear model with negative binomial distribution was used to test the effect of dietary treatment on fledging age.

We tested for possible relationships between fledging age and several other variables, including concentrations of ALA and AA in RBCs at 36 dph, the average activity and stress behaviours recorded at 35 and 37 dph, and the mass gained from hatching to 36 dph. Each predictor was tested using a separate generalized linear regression with a negative binomial distribution to address overdispersion. Upon finding a significant relationship between fledging age and ALA, we further investigated the relationship by comparing the concentration of ALA in the RBCs at 36 dph among the three dietary treatments using a linear model. We also tested for a relationship between the concentration of DHA in the blood and the concentration of its precursors ALA and EPA in the blood at 36 dph using a linear model.

## RESULTS

### Incorporation of n-3 LCPUFAs into RBCs and brain tissue

**Fig. 2.**
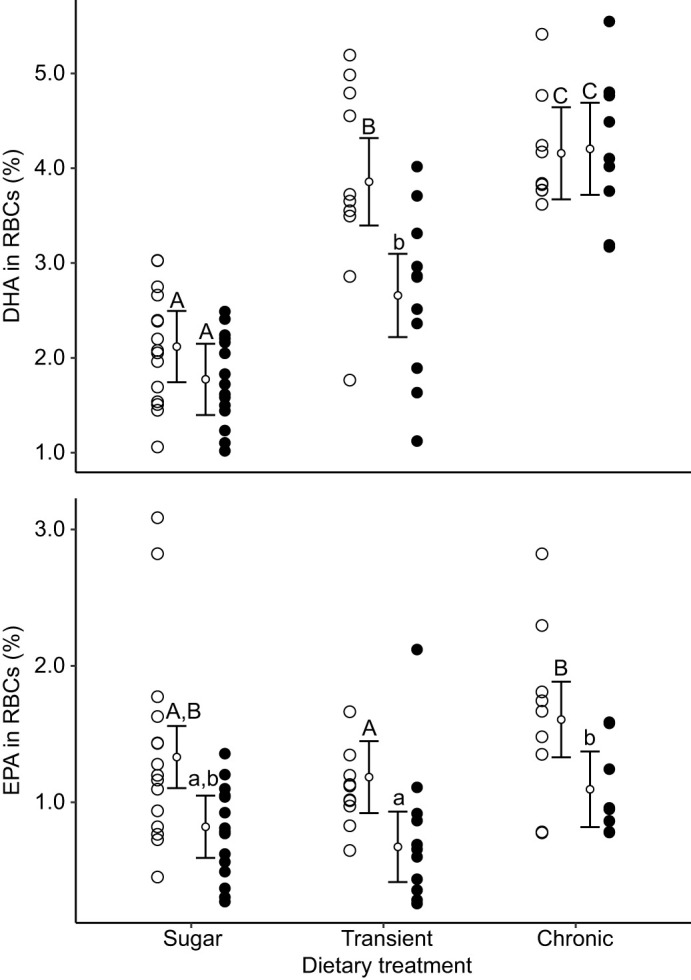
**Incorporation of omega-3 fatty acids (n-3 LCPUFAs) into red blood cells (RBCs).** The concentration of two n-3 LCPUFAs (docosahexanoic acid, DHA; eicosapentaenoic acid, EPA) was measured in the RBCs of 36 chicks at 15 (open circles) and 36 days post-hatching (dph; filled circles). Values are expressed as relative concentration (percentage of total identified fatty acids) and compared among three dietary treatments: chronic oil, transient oil and sucrose solution control. Each mean (small circle) is presented with its 95% confidence interval and raw data (large circles). Age groups within a treatment are significantly different when the letters above their confidence intervals are of different case, and treatment groups are significantly different when they do not share the same letters (Tukey *post hoc* tests).

Chicks supplemented with fish oil each day between hatching and fledging (chronic oil treatment) had significantly higher levels of DHA in their RBCs than chicks that were supplemented with a sucrose solution control during the same period, as revealed by the analysis of blood samples taken at 15 and 36 dph (Table 1, Fig. 2). Chicks that were supplemented with fish oil for 22 dph, but then switched to the sucrose solution control until fledging (transient oil treatment), had DHA levels comparable to those of the chronic oil group at day 15. At day 36, DHA in the transient oil treatment had declined relative to levels at day 15, and relative to the chronic oil treatment, but were still higher compared with the control treatment. These effects were revealed through statistically significant effects of treatment, age, and the interaction between treatment and age (Table 1, Fig. 2). The concentration of EPA in RBCs was significantly higher in the chronic oil group than in the transient oil group, but neither group differed significantly from the sucrose solution control group (Table 1, Fig. 2). EPA decreased significantly between 15 and 36 dph, with no interaction between age and dietary treatment (Table 1, Fig. 2).

**Table 1.**
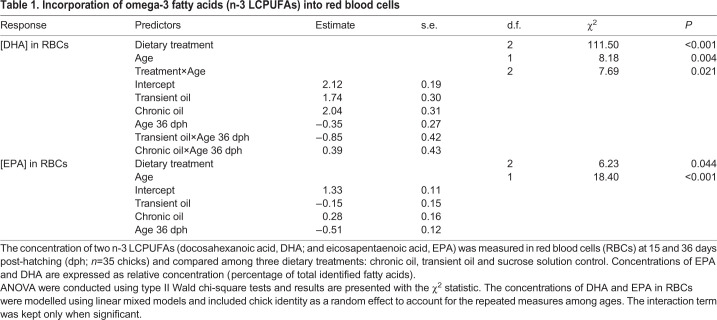
**Incorporation of omega-3 fatty acids (n-3 LCPUFAs) into red blood cells**

**Fig. 3.**
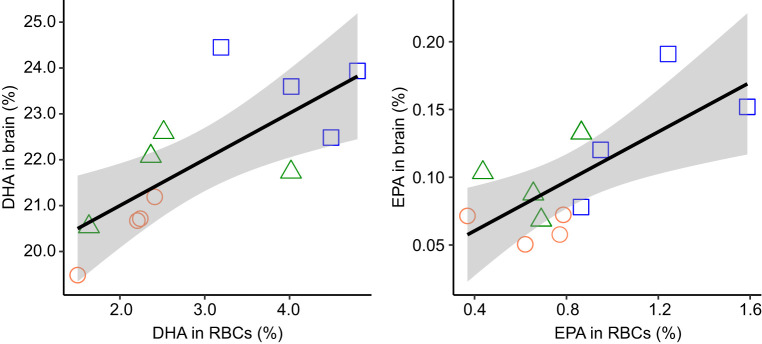
**Incorporation of n-3 LCPUFAs into RBCs predicts incorporation of n-3 LCPUFAs into the cerebral hemispheres of the brain.** The concentrations of two n-3 LCPUFAs (DHA and EPA) were measured from the RBCs of 12 chicks at 36 dph and from the cerebral hemispheres of the same chicks at 42 dph, and are expressed as relative concentration (percentage of total identified fatty acids). The relationships (±s.e.) between the concentrations of n-3 LCPUFAs in both tissues, as predicted by our models, are represented by a black line (with grey shading). Raw data are represented by the points, with colours and shapes corresponding to the treatment groups (orange circles, sucrose solution control; green triangles, transient oil; blue squares, chronic oil; *n*=4 for each).

The concentration of n-3 LCPUFAs in RBCs was positively correlated with the concentration of n-3 LCPUFAs in the brains of a subset of 12 chicks (4 per dietary treatment) that were removed at 42 dph for brain tissue analysis (Fig. 3). The concentration of DHA in the cerebral hemispheres increased significantly with increases in the RBCs, at a rate of 1:1 beyond the intercept (linear regression: *F*_1,10_=12.27, *P*=0.006, *R*^2^=0.55, *y*=1.01*x*+18.99; Fig. 3). The concentration of EPA in the cerebral hemispheres also increased significantly with increases in RBCs, though the rate of increase was less pronounced than for DHA (*F*_1,10_=10.58, *P*=0.009, *R*^2^=0.51, *y*=0.09*x*+0.02; Fig. 3). As the concentration of n-3 LCPUFAs in RBCs reflects the concentration of n-3 LCPUFAs in the brain, it was used in subsequent analyses owing to the larger sample size, repeated measures and the possibility of comparing it with fledging age. The fatty acid composition of RBCs and cerebral hemispheres for each dietary treatment group is provided in Table S3.

### Chicks with more DHA fledge earlier

**Fig. 4.**
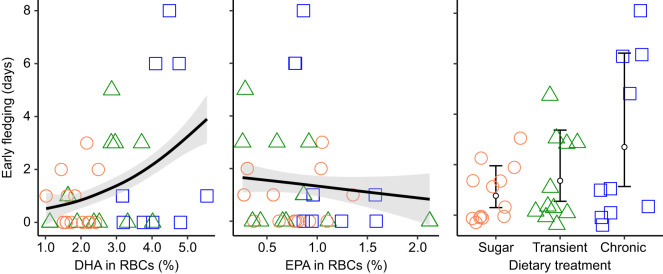
**Chicks with more DHA fledged earlier.** Fledging age was measured as the number of days before 42 dph, which was assigned as the maximum age of fledging in our study. Concentrations of two n-3 LCPUFAs (DHA and EPA) were measured in RBCs at 36 dph and expressed as relative concentration (percentage of total identified fatty acids). This model is based on a sample size of 33 chicks, as two were attacked and died in their nest before fledging (38 dph for both) and one from the chronic oil treatment fledged early at 34 dph and by 37dph but could not be captured for a blood sample at 36 dph. The predicted relationships (±s.e.) are represented by a black line (with grey shading). Treatment, which was analysed separately from the concentrations of DHA and EPA in the RBCs (right panel), did not have a significant effect on fledging age (*n*=34 chicks). Each mean (small circle) is presented with its 95% confidence interval. Raw data are represented by the points, with colours and shapes corresponding to the treatment groups (orange circles, sucrose solution control *N*=13; green triangles, transient oil *N*=11; blue squares, chronic oil *N*=9 for the DHA+EPA model, *N*=10 for the treatment model).

Although dietary treatment was not a significant predictor of early fledging, the concentration of DHA in chick RBCs at 36 dph was. Thus, chicks with a greater DHA concentration in their blood fledged significantly earlier than chicks with less DHA, regardless of supplementation (Table 2, Fig. 4). In contrast, the concentration of EPA in the blood at 36 dph was not significantly related to fledging age (Table 2, Fig. 4).

**Table 2.**
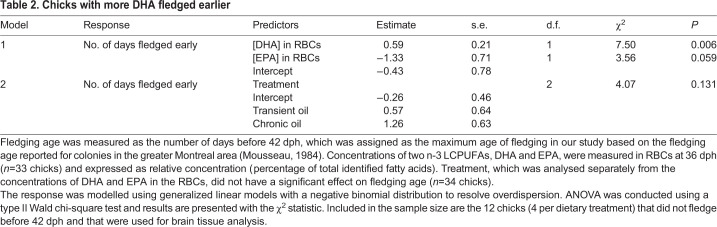
**Chicks with more DHA fledged earlier**

### Further exploration of the relationship between DHA and fledging age

**Fig. 5.**
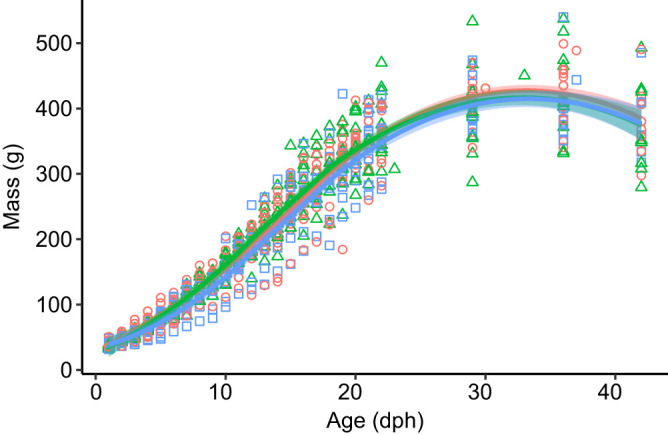
**Mass gained during the nestling period in the three dietary treatment groups.** Growth was measured by weighing the chicks (*N*=10 chronic oil, *N*=11 transient oil, *N*=15 sucrose solution control) daily from hatching until 23 dph and weekly afterwards until fledging. The mean growth trajectories are represented by the lines (±s.e. shading), modelled using the average non-parametric local weighted regression of each dietary treatment group. Raw data are represented by the points, with colours and shapes corresponding to the treatment groups (orange circles, sucrose solution control; green triangles, transient oil; blue squares, chronic oil). The linear growth phase occurred between 5 and 22 dph and the growth curves appear identical to growth curves of unsupplemented ring-billed gulls published in previous studies (Chardine, 1978; Dawson et al., 1976; Iacovides and Evans, 1998; Oswald et al., 2013).

**Fig. 6.**
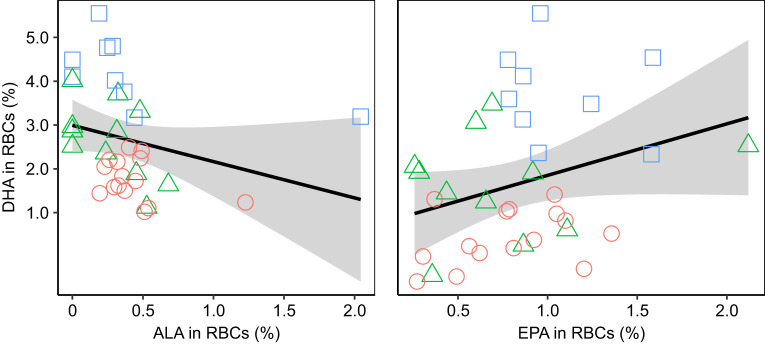
**DHA in RBCs is negatively related to** **ALA** **and positively related to EPA.** The concentrations of DHA, α-linolenic acid (ALA) and EPA were measured in RBCs at 36 dph (*n*=35 chicks, as one chick from the chronic group fledged before a blood sample could be taken at 36 dph) and expressed as relative concentration (percentage of total identified fatty acids). The predicted relationships (±s.e.) are represented by a black line (with grey shading). Raw data are represented by the points, with colours and shapes corresponding to the treatment groups (orange circles, sucrose solution control *N*=15; green triangles, transient oil *N*=11; blue squares, chronic oil *N*=9).

We could not use the chicks' ability to solve the string-pull test as a direct measure of their cognitive performance because chicks generally did not attempt to solve the test. The role of cognition in early fledging therefore remains unclear. However, we tested several alternative hypotheses that could potentially explain the relationship between DHA and fledging age. First, it is possible that chicks receiving fish oil fledged earlier if the oil accelerated growth. However, the mass gained by chicks between hatching and 36 dph was unrelated to fledging age (Table 3, Fig. 5). Second, chicks receiving fish oil could have fledged earlier if the oil caused them to be generally more active or to spend more time in the centre of the enclosure, where it would be easier to fly up and over the fence. However, general activity levels and time spent touching the fence in the context of the string-pull tests had no relationship with fledging age (Table 3). Third, high levels of n-3 LCPUFAs, such as those observed in experimental chicks, can counteract the natural accretion of AA in tissues (de Haas et al., 2017; Drover et al., 2011; Speake et al., 2003), which is also necessary for optimal cognitive development in vertebrates (de Haas et al., 2017; Hadley et al., 2016; Marszalek and Lodish, 2005). However, the concentration of AA in RBCs at 36 dph was high in all dietary treatments (Table S3), presumably as a function of parental provisioning, and was unrelated to fledging age (Table 3). The relationship between DHA and fledging age thus cannot be explained by mediating effects of mass, behaviour or AA. In contrast, the concentration of ALA in RBCs at 36 dph was inversely related to fledging age (Table 3). To better understand the relationship between ALA and fledging age, we compared ALA among dietary treatments and we tested for a possible relationship between DHA and its precursors ALA and EPA. We found that ALA did not differ among experimental treatments. However, the concentration of DHA was inversely related to the concentration of ALA and positively related to the concentration of EPA in the RBCs at 36 dph (Table 4, Fig. 6).

**Table 3.**
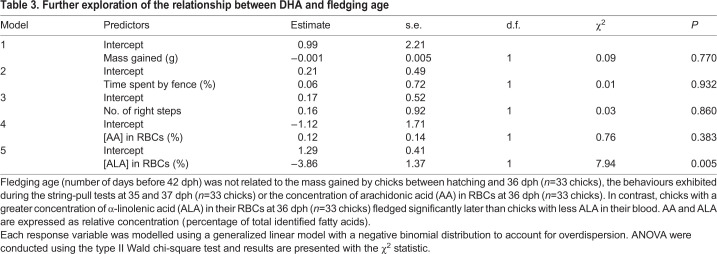
**Further exploration of the relationship between** **DHA and fledging age**

**Table 4.**
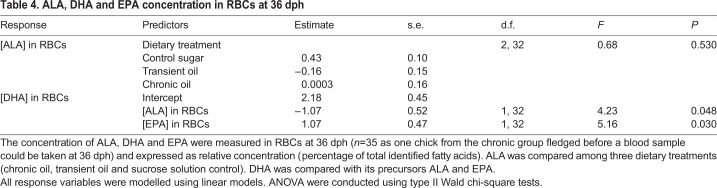
**ALA, DHA and EPA concentration in RBCs at 36 dph**

## DISCUSSION

We investigated the positive effect of n-3 LCPUFA supplementation on the cognition of ring-billed gull nestlings in a population feeding primarily on terrestrial food sources with access to little to no n-3 LCPUFAs. We showed that fish oil supplementation increased the concentration of DHA in the RBCs and cerebral hemispheres of chicks. Furthermore, DHA levels in RBCs quickly returned to control levels in chicks where fish oil supplements were suspended at 22 dph, and DHA levels in the brains of those chicks became intermediate between those of chicks in the control and chronic oil treatment groups at fledging. Fledging occurred earlier among chicks with more DHA; this relationship could not be explained by differences in activity levels, time spent by the fence, body mass or AA levels in the RBCs. Levels of ALA in RBCs, however, covaried with levels of DHA and with fledging age, suggesting a possible mediating role.

Chicks with more DHA in their RBCs at 36 dph fledged earlier than chicks with less DHA in their RBCs. Previous studies have shown that n-3 LCPUFA supplementation accelerates growth in tree swallows (*Tachycineta bicolor*; Twining et al., 2016) and eastern phoebes (*Sayornis phoebe*; Twining et al., 2019), as compared with control chicks given supplements with equal calories but no n-3 LCPUFAs. In our study, however, body mass gain did not correlate with fledging age and thus cannot explain the relationship between fledging age and DHA. Similarly, previous studies have demonstrated that body growth and the accretion of encephalic DHA occur independently of each other in mammals and birds (Hulbert et al., 2002; Speake and Wood, 2005). Speake and Wood (2005) hypothesize that chicks can only fledge once they have accumulated enough DHA in their brains to reach sufficient cognitive maturation, even if they have attained sufficient skeletal and muscular maturation (also supported by Thil et al., 2003). This hypothesis derives partially from the observation that precocial species accumulate DHA in their brains pre-hatching and gain some autonomy shortly after hatching, whereas altricial species accumulate most of their DHA post-hatching and gain autonomy only prior to fledging (Speake and Wood, 2005). Furthermore, an observational study (Dodson et al., 2016) found that altricial prothonotary warblers (*Protonotaria citrea*) fledged earlier when they were fed aquatic insects versus terrestrial insects, despite the two groups showing similar body condition. Although the n-3 LCPUFA content of the insects was not analysed in that study, the authors speculated that chicks feeding on aquatic insects consumed more n-3 LCPUFAs and, consequently, fledged earlier (Dodson et al., 2016). Ring-billed gulls, as semi-precocial seabirds (Pollet et al., 2012), are expected to attain adult-like levels of encephalic DHA quickly, as long as their consumption of n-3 LCPUFAs pre- and post-hatching is optimal (Speake and Wood, 2005). We suggest that chicks supplemented with fish oil in our study accumulated an optimal amount of DHA in their brains sooner than chicks that did not receive fish oil, thereby accelerating the cognitive maturation necessary for fledging. In contrast, chicks in our control group likely had suboptimal intake of n-3 LCPUFAs pre- and post-hatching because their parents fed primarily on terrestrial and anthropogenic diets with little to no n-3 LCPUFAs (Caron-Beaudoin et al., 2013; Marteinson et al., 2015).

The accretion of DHA in the brains of semi-precocial ring-billed gull chicks continued to increase beyond hatching, and beyond their linear growth phase (5–22 dph; Figs 2, 3, 5). This contrasts with previous research showing that the concentration of DHA in the brains of wild precocial and semi-precocial species is mainly influenced by the quantity of n-3 LCPUFAs present in the yolk, and that it remains constant between hatching and adulthood (Speake and Wood, 2005; Speake et al., 1996, 2003; Surai et al., 2000). Our finding can be explained by the plasticity exhibited by neuronal tissues when sub-optimal nutrition is provided to a developing individual (Diau et al., 2005; Fan et al., 2016; Ikemoto et al., 2001), as was the case for chicks in our control treatment. Other studies of birds with dietary n-3 LCPUFA deficiency, due to being fed a captive diet, foraging on anthropogenic food sources, or being deprived of preferred aquatic prey, have also been able to increase n-3 LCPUFAs in chick brains through fish oil supplementation (e.g. domestic chicken: Poureslami et al., 2010; tree swallows: Twining et al., 2016; Muscovy ducks, *Cairina moschata*: Baéza et al., 2017; red-winged blackbirds: Price et al., 2018). Indeed, many authors have remarked that captive and anthropogenic diets are deficient in n-3 LCPUFAs, but that the introduction of n-3 LCPUFAs in the animal's diet could remedy the deficiency to a certain degree (Clauss et al., 2007; Maldjian et al., 1996; Simopoulos, 1999; Surai et al., 2001). This explains why DHA in these avian brains could be manipulated through dietary supplementation of n-3 LCPUFAs post-hatching.

Our observation that chicks with more DHA fledge earlier is consistent with our hypothesis that DHA improves cognition, which, in turn, helps chicks solve problems such as escaping the fence encircling their nest. Our argument that flying over the fence at a younger age reflects enhanced cognition is based on previous studies. Thorndike (1898) was the first to suggest that the ability to escape a box could reflect problem-solving skills in animals, including in cats (*Felis catus*), dogs (*Canis lupus familiaris*) and chickens (*Gallus gallus domesticus*). Since then, more studies have measured animals' cognitive performance through their ability to escape from a puzzle box (Cauchard et al., 2013; Galsworthy et al., 2005; Grundy et al., 2014). Furthermore, because we constructed the fences out of semi-transparent burlap, we suggest that flying over the fence (fledging) approximated an obstacle detour task, where subjects must access a reward placed behind a transparent barrier by choosing a non-obvious route; successful completion of the task implies self-control, planning and memory (Kabadayi et al., 2018; MacLean et al., 2014; Regolin et al., 1995; Zucca et al., 2005). Indeed, as soon as chicks were mobile, they all tried unsuccessfully to get through the fence by pushing on the burlap whenever we approached their nest. Thus, chicks could only fledge once they abandoned this strategy and, instead, attempted to leave the nest by flying upwards and away from neighbouring gulls. Nonetheless, we cannot exclude the possibility that DHA led to early fledging through non-cognitive means. For example, consuming n-3 LCPUFAs leads to enhanced muscular aerobic performance in certain bird species (Guglielmo, 2010; Maillet and Weber, 2007; Nagahuedi et al., 2009). However, we think this is unlikely because DHA's role in muscles appears restricted to helping refuel muscles during long flights and does not increase muscle strength or accelerate muscle development (Dick and Guglielmo, 2019; Keegan et al., 2019; Price and Guglielmo, 2009; Yan and Kim, 2013), which we suggest would be necessary for flying over the fences at an earlier age. Therefore, we cautiously suggest that DHA, given its accretion in chick brains based on n-3 LCPUFA supplementation, was responsible for their early fledging, although more studies are required to thoroughly explore the link between n-3 LCPUFAs and avian cognition.

In contrast, variation in fledging age could not be predicted by several other factors. For example, the behaviour of chicks (activity level and time spent next to the fence) did not explain their younger fledging age. This was important to investigate because n-3 LCPUFA supplementation can affect stress behaviours (Aigueperse et al., 2013; Baéza et al., 2017; de Haas et al., 2017; Fedorova and Salem, 2006; Kuratko et al., 2013; Vinot et al., 2011), which could facilitate early or accidental fledging. A chick prone to moving, or one that spends more time in the centre of the enclosure, could escape the fence sooner simply as a result of spending more time in the right place, as opposed to intuiting a solution. Likewise, the level of AA had no influence on the chicks' ability to fledge early, despite being high in all dietary groups. AA facilitates neuronal growth and signalling (Marszalek and Lodish, 2005; Speake et al., 1998), and variation in its concentration can affect the rate of cognitive development (review by Hadley et al., 2016). AA and n-3 LCPUFAs compete metabolically against each other, leading to a reduction in the absorption and action of the less abundant one (Brenna et al., 2009; Saini and Keum, 2018). It is therefore possible that AA disproportionately suppressed DHA in chicks that did not receive fish oil supplements, which may have strengthened the relationship between DHA and cognition and, ultimately, the observed relationship between DHA and fledging age. Given the lack of treatment group differences in growth trajectory and activity levels, we are confident that our choice of sucrose as a control neither positively nor negatively impacted our control and transient chicks. However, we cannot rule out the possibility that the disparity in the macronutrient composition of fish oil and sucrose influenced our results beyond the mere presence or absence of n-3 LCPUFAs. Consequently, future studies would benefit from using microencapsulated fatty acids as a way to test the specific effects of n-3 LCPUFAs on avian cognition. These products are designed to prevent oxidative damage and, thus, can be given at the same melting temperature as liquified saturated fats (Fard et al., 2020; Jiménez-Martín et al., 2015; Xia et al., 2019), which are often the standard controls in fatty acid studies.

Chicks with more ALA in their RBCs fledged later than chicks with less ALA. Furthermore, the concentrations of EPA and DHA were positively related in the blood of chicks, as expected given they were both present in the fish oil supplement (Table S1), but the concentrations of ALA and DHA were inversely related despite no difference in ALA among dietary treatment groups (Table 4). This inverse relationship between DHA and its precursor has been reported in previous studies where a high consumption of ALA inhibited the endogenous conversion of ALA to DHA by preferentially binding to the enzyme needed to produce DHA (Geiger et al., 1993; Gibson et al., 2011; Li et al., 2000). It is unknown whether such inhibition could occur in species relying on the dietary consumption of DHA because inhibition has only been documented in species that retain the ability to convert ALA into EPA and DHA, and only when those species were supplemented with ALA in the absence of n-3 LCPUFAs (humans, *Homo sapiens*: Chan et al., 1993; pigs, *Sus domesticus*: Blank et al., 2002; rats, *Rattus norvegicus domestica*: Gibson et al., 2013; Tu et al., 2010; barramundi, *Lates calcarifer*: Tu et al., 2013). It remains unknown whether ring-billed gulls can convert any dietary ALA into DHA, though this ability has been documented in other omnivorous and generalist species, even though their conversion is inefficient (Castro et al., 2012; Martinez del Rio and McWilliams, 2016; Twining et al., 2018b; Gladyshev and Sushchik, 2019). If they could convert ALA provisioned to them by their parents into DHA, then higher ALA intake might have inhibited DHA synthesis, thus explaining our results. Given that we detected only trace levels of ALA (<0.5%) in all bird tissues (Table S3), this explanation should be considered with caution. Future research should investigate the ability of aquatic avian predators and generalists to endogenously synthesize n-3 LCPUFAs from ALA.

Although EPA and DHA were positively associated, the concentration of EPA in RBCs and brain tissue was low in all chicks and did not differ significantly among dietary treatment groups, even though its concentration in the Menhaden fish oil supplement was higher than that of DHA (Table S1). Previous fish oil supplementation studies of chicks and young mammals (Anderson et al., 1989; Cherian, 2015; Twining et al., 2016, 2019) show that the concentration of EPA in the blood and brain increases only slightly, even if the animal consumes extremely high amounts of EPA (Chen et al., 2009; Saini and Keum, 2018; Stark et al., 2016). The disconnect between the amount of EPA ingested and the amount in the blood is thought to be due to the rapid conversion of EPA to other metabolites (primarily eicosanoids and DHA; Anderson et al., 1989; Chen et al., 2013; Kaur et al., 2010; McNamara and Carlson, 2006) and to the poor incorporation of EPA into cell membranes (Chen et al., 2013; McNamara and Carlson, 2006). Although both EPA and DHA supplements provide neurological benefits, such as enhanced synaptic plasticity and encephalic perfusion (Bazinet and Layé, 2014; Calder, 2015; Yang et al., 2018), most studies show that only the accretion of DHA in tissues enhances cognition (Gale et al., 2008; Janssen et al., 2015; Joffre et al., 2014; Mulder et al., 2014; Saini and Keum, 2018; Weiser et al., 2016).

Early fledging as a result of rapid accretion of encephalic DHA could provide several benefits in birds, whereas delayed fledging due to dietary deficiencies in DHA could have the opposite effect. For species experiencing high nest predation, fledging as little as 1 day early improves a chick's survival by decreasing its time in the nest, where it is most vulnerable to predation (willow warbler, *Phylloscopus trochilus*: Bjørnstad and Lifjeld, 1996; prothonotary warbler, *Protonotaria citrea*: Dodson et al., 2016). In murres (*Uria* spp.), there is strong selection for later-hatched chicks to develop up to 5 days faster so that they fledge during the colony's peak fledging period (Benowitz-Fredericks and Kitaysky, 2005; Hatchwell, 1991) and benefit from the associated predator swamping effect (Gaston and Nettleship, 1981; Hatchwell, 1991). In avian species where territoriality or social dominance occurs, juveniles that fledge earlier can gain higher social rank than older individuals that fledge later (herring gulls: Nisbet and Drury, 1972; black-capped chickadee, *Poecile atricapillus*: Glase, 1973; western gulls, *Larus occidentalis*: Briggs, 1978; marsh tit, *Parus palustris*: Nilsson, 1990; willow tit, *Parus montanus*: Thessing, 1999; European shag, *Phalacrocorax aristotelis*: Velando, 2000; black-headed gull, *Larus ridibundus*: Prévot-Julliard et al., 2001). Indeed, Canada jays (*Perisoreus canadensis*) gain territoriality benefits if they fledge 15–30% earlier than their typical fledging age of 22–24 days (Freeman et al., 2020). In species where dispersal is delayed, younger fledglings can benefit by having more time to explore their surroundings while still benefiting from the protection conferred by their colony or parents (Franklin's gulls, *Larus pipixcan*: Burger, 1972; herring gulls: Burger, 1981; sparrowhawks, *Accipiter nisus*: Frumkin, 1994; sooty terns, *Sterna fuscata*: Feare, 2002). Gulls that fledge earlier in the season also have an increased probability of surviving the juvenile phase and a higher likelihood of successfully reproducing once they reach adulthood (Franklin's gulls: Burger, 1972; herring gulls: Nisbet and Drury, 1972; Parsons et al., 1976); glaucous-winged gulls, *Larus glaucescens*: Hunt and Hunt, 1976; western gulls: Spear and Nur, 1994; black-headed gulls: Prévot-Julliard et al., 2001). In the Nazca booby (*Sula granti*), a long-lived seabird that fledges at around 157 days old, every 1 day increase in fledging age reduces the probability of surviving the juvenile phase by 3% (Maness and Anderson, 2013). In seabirds, a rapid decrease in parental provisioning as chicks approach their typical fledging age causes a drop in nestling body condition and thus encourages early fledging (Graves et al., 1991; Ritz et al., 2005). Finally, fledging early can also benefit a chick's parents. In puffins and auklets (family Alcidae), for example, parents experience high predation risk at the nest (e.g. puffins: Harris, 1980; auklets: Harfenist, 1995; Nelson, 1989) and may thus have better odds of survival if their chicks fledge early. In murres, where parental provisioning continues long after fledging, parents of chicks that fledge early conserve energy by not having to fly back and forth to the colony to provision their chick (Birkhead, 1977; Ydenberg et al., 1995). It is unclear whether earlier fledging provides any or all of these benefits in ring-billed gulls, but these studies, combined with our own, suggest that delayed fledging caused by environmental deficiencies in n-3 LCPUFAs could have multiple negative impacts on diverse avian taxa.

Our study shows that developing ring-billed gulls from a population with restricted access to dietary n-3 LCPUFAs fledge earlier when supplemented with n-3 LCPUFAs versus a sucrose solution control. We argue that the supplemental n-3 LCPUFAs improved their cognition, which, in turn, allowed them to escape the fence encircling their nest and fledge earlier. In birds, cognitive ability generally correlates with a species' ability to learn, innovate, locate food, avoid predators and adapt to novel or changing environments (Timmermans et al., 2000; Lefebvre et al., 2016; Ducatez and DeVore, 2019). With 70% of seabird species declining worldwide (Paleczny et al., 2015), a reduction in dietary n-3 LCPUFAs in aquatic ecosystems as a result of warming and acidification may thus impair the cognition of seabirds, but also of cetaceans, pinnipeds and other aquatic predators that consume diets historically rich in n-3 LCPUFAs (Colombo et al., 2020), and thus compromise their ability to solve novel real-world challenges and cope in a rapidly changing world (Beaugrand et al., 2019; Lovenduski et al., 2019).
